# Whooping-Cough of Infants, and Treatment to Prevent Complications

**Published:** 1900-02

**Authors:** C. E. Murphey

**Affiliations:** Atlanta, Ga.


### WHOOPING-COUGH OF INFANTS, AND TREATMENT TO PREVENT COMPLICATIONS.*

BY C. E. MURPHEY, M.D.,

ATLANTA, GA.

Mr. President, I almost feel as if I should apologize for bringing this subject—one of the most common and oldest diseases known

*Read before the Atlanta Society of Medicine.

to mankind—before this Society of Medicine, but my experience with whooping-cough of late has been of such a character that I would be glad to hear the subject discussed before this Society.

While the advancement in the science of medicine has been great in many lines, I am sure there has been but little progress in the care and treatment of pertussis, and many other of what are considered the older contagious diseases.

I will not go into the definition, history, etiology, or pathology of pertussis, but will only take up a few practical points. I am aware of the fact that there is no disease of childhood that the general practitioner of medicine more often comes in contact with than whooping-cough, but I am also of the opinion that there is no disease more often neglected, with fatal results, than the one just mentioned. The parents pay but little attention to children with whooping-cough, because it is a popular idea that there is no danger and that the disease will have to wear itself out. Physicians are largely responsible for this false idea. A mother will come to you stating that "my children have all had whooping-cough, but it has gone to my baby's lungs, and I want to know what best to do." Next thing you hear is a funeral.

I think it just as important to isolate whooping-cough from infants—delicate children, those with a predisposition to tuberculosis—for it is with them chiefly that we are likely to have trouble, as it would be in a case of scarlet fever.

Infants especially are more likely to take on fatal complications than older children; therefore, mothers must be taught the danger in pertussis, so that common-sense principles may guide her to keep a child indoors if weather is unfavorable, and isolate her infant or delicate children if such are in her family.

The complications in pertussis are the serious part we will have to deal with. Therefore every precaution should be exercised to prevent these complications.

The most serious of these complications to infants are connected with the lungs, and usually broncho-pneumonia. This is especially to be dreaded in infancy. Convulsions are also more frequent in infants. Bronchitis of the large tubes, which sometimes proves to be a source of trouble for some months. Hemorrhage sometimes occurs, depending upon intense venous congestion following severe paroxysms. These are the most prominent complications in infancy, of which a large per cent. die.

As for the care and treatment of pertussis, the general hygienic condition of the household is an important factor, as we all know diseases seek dirt; that foul air and sewer gases are the favorite elements in which this as well as other contagion thrives. I think the day will come when the general practitioner of medicine will care for these contagions as the "nowaday" surgeon does in his operative work. The rooms should not only be kept cleanly, but well ventilated. Children affected must have plenty of fresh air, and, if the weather will permit, should be kept out to breathe pure atmospheric air. Oxygen is a great destroyer of contagion, and one of the greatest disinfectants. Observation has proven that children have fewer paroxysms while out of doors than in the house, and that the paroxysms are very much more frequent when confined in close rooms. It is important to keep the rooms well ventilated at night, unless bronchitis or broncho-pneumonia are present. I think rooms occupied by children should frequently be changed, thoroughly aired. I also think it well to disinfect the rooms by burning ordinary sulphur or other disinfectant—of course removing the children from the room beforehand.

The scientific physician does not pretend to cure whooping-cough, and many of the specifics introduced claiming the power of arresting the disease are worthless and have fallen into disuse. As in scarlet fever, typhoid fever, and many other contagions, it will run its course, but as physicians we strive to guide the course of the disease, avoiding complications, and thereby pilot our little patients safely to land. There is but little treatment needed by the use of drugs in the first stage. We must remember the leading indications are to allay irritation. Therefore, in the first stage, a simple mixture of ipecac, squills, liquor potassi citrate will be of great service. When the first stage is passed and that characteristic paroxysm of coughing is well established, I strongly recommend belladonna. We can also call to our aid wine of ipecac, syr. squill and carbonate potass. Belladonna appears to relieve spasm, cut short the paroxysm, assist expectoration and relieve the irritability of the lungs. I also think well of antikamnia with bromide of soda, but in the event of pneumonia developed the antikamnia should be discontinued. I have seen excellent effect produced by chloral hydrate, of which the youngest infants are very tolerant, and especially is the chloral beneficial where the night attacks are so severe as to prevent sleep. An excellent combination is chloral and bromide of soda. It takes less of the chloral to produce the required impression when given with the bromide. I want to mention one case in which chloral hydrate did great work for me.

Fannie B., aged three years, entering the fourth week of pertussis. Everything she would take brought on a paroxysm, even the small amount of water she drank. The mother had discarded medicine, water, food, in fact, allowed nothing to go in her stomach on account of severe paroxysm which followed. She stated that the child had slept but three hours for the past two nights on account of the paroxysms of coughing. I gave the child eight grains of chloral hydrate by rectum, and in one hour gave six more. The little patient slept for four hours without waking. On waking she had a light paroxysm, and again slept for an hour and a half. I then gave her the same amount of chloral (eight grains) and requested the mother to arouse her in half an hour and give her half a glass of milk with teaspoonful of whiskey, which she retained nicely. I kept up this treatment for several days. The child retained its nourishment, slept well, and of course the paroxysms were far apart. I think in this case the child's life was saved. The chloral not only produced the much-needed sleep, but relieved that nervous condition that caused the frequent paroxysms and vomiting and sometimes convulsions.

In speaking of belladonna I failed to state that it should be given in small doses, repeated until the physiologic effect is produced, after which give a few drops less, and in this way you can continue for any length of time. I use none but Squibbs's belladonna, either the tincture or extract. If you employ an inferior quality of belladonna then you produce no impression whatever on your patient, therefore unjustly discard the remedy as useless.

I have used antiseptic agents, the best of which are the vapors of carbolic acid, cresolin and thymol, with apparent good results.

To say how many remedies I have used with no results would likely fill many pages; therefore I'll mention none of those.

I will not go into treatment of the many complications, as I only wanted to speak of the care and treatment of pertussis to prevent such complications.

---

### EXCISION OF HEMORRHOIDS.

By G. Reinbach. The author reports eighty-one cases of hemorrhoids operated upon in Mikulicz's clinic, in Fränkel's clinic, and in the Augusta Hospital in Breslau. The usual method followed was that advocated by Whitehead in 1887. Mikulicz modifies this to some extent: he does not dilate the sphincter, he ligates the bleeding vessels with catgut in place of silk, irrigation is used to keep clean the area of operation instead of sponges. The thermocautery, after the method of v. Langenbeck, was only used in cases in which there was ulceration of the skin or mucous membrane, abscesses, or such inflammatory appearances as phlebitis and periphlebitis. In all other cases excision was used. As advantages of the latter procedure, the author claims that the technic is simple, that all the affected tissue may be removed, that the formation of a stricture is impossible, that it is particularly free from danger, as primary union is obtained and hence infection and secondary hemorrhage avoided. These latter may complicate the operation by the thermocautery, as there a scab is cast off. The final results favor excision. All the cases were discharged from the hospital healed. None were infected. Secondary hemorrhage occurred in but three cases. The sutures gave way extensively in but one case. Of seventy cases which could be followed up, sixty-nine were permanently cured. On the other hand, of twenty-seven cases treated by means of the thermocautery, there were four probable recurrences and one certain recurrence. Consequently the author advocates excision rather than the thermocautery.—*Beiträge zür klin. Chirurgie*, Bd. XXIII., Hft. 3.—*Brooklyn Med. Journal.*

